# Case Report: Atypical pediatric allergic bronchopulmonary aspergillosis masquerading as recurrent pneumonia

**DOI:** 10.3389/fped.2026.1795020

**Published:** 2026-05-18

**Authors:** Jingjing Ying, Jin Chen, Danhong Fei, Yongjun Dai, Cheng Zheng

**Affiliations:** 1 Department of Pediatrics, Taizhou Municipal Hospital (Taizhou University Affiliated Municipal Hospital), School of Medicine, Taizhou University, Taizhou, China; 2 Department of Critical Care Medicine, Taizhou Municipal Hospital (Taizhou University Affiliated Municipal Hospital), School of Medicine, Taizhou University, Taizhou, China; 3 Taizhou Key Laboratory of Infection and Tumor Immunology, Taizhou Municipal Hospital, Taizhou, China

**Keywords:** allergic bronchopulmonary aspergillosis, *Aspergillus fumigatus*, difficult-to-treat asthma, pediatric, recurrent pneumonia

## Abstract

Allergic bronchopulmonary aspergillosis (ABPA), a hypersensitivity lung disease mediated by *Aspergillus fumigatus*, represents a frequently overlooked diagnosis in pediatric practice. We describe an 11-year-old girl with difficult-to-treat asthma and recurrent pneumonias (three episodes within one year) in whom comprehensive evaluation revealed borderline total IgE (678 IU/mL), *Aspergillus*-specific IgE of 5.81 kUA/L, high-resolution CT demonstrating bilateral infiltrates with scattered nodules, and *Aspergillus fumigatus* identification via bronchoalveolar lavage fluid culture and targeted next-generation sequencing (tNGS). Initiation of systemic corticosteroids plus itraconazole produced rapid clinical resolution within three weeks, decreased total IgE by 29% (to 479 IU/mL) at one month, and achieved marked radiographic improvement by four months. This case highlights the need to consider ABPA in children with difficult-to-treat asthma and recurrent pulmonary infections, suggests *Aspergillus*-specific IgE screening in difficult-to-treat asthma, while diagnosis should rely on integrated clinical, immunologic, radiologic, and microbiologic assessment.

## Introduction

Allergic bronchopulmonary aspergillosis (ABPA), an immunologically mediated hypersensitivity lung disease triggered by *Aspergillus fumigatus* airway colonization, constitutes a severe and chronically underrecognized complication of both asthma and cystic fibrosis (CF). The global ABPA burden among asthma populations is estimated at 4.8–5.0 million cases, with India alone accounting for 1.4 million (1). However, diagnostic recognition remains strikingly inconsistent: a meta-analysis of tertiary-care adult asthma patients documented a pooled prevalence of 11.3%, with Indian cohorts representing the sole geographic predictor of elevated risk (2). Among children, pooled prevalence reaches 9.9% in asthmatic populations, yet remarkably, no cases were reported from the United Kingdom or United States in this systematic review (3). Most troubling, direct epidemiological data for Chinese pediatric populations remain entirely absent, creating a critical evidence void in the context of China's substantial childhood asthma burden.

Widespread underdiagnosis in clinical practice compounds this lack of epidemiological data. Pediatric ABPA has only gained significant recognition in the past decade, highlighting persistent gaps in clinical awareness and systematic screening (4). This diagnostic delay has serious consequences: ABPA is frequently misdiagnosed as bacterial pneumonia, resulting in unnecessary antibiotic use, delayed appropriate treatment, and progressive lung damage that may become irreversible.

We herein report an instructive case of an 11-year-old girl with difficult-to-treat asthma who experienced three misdiagnosed pneumonia episodes within one year. Her eventual ABPA diagnosis highlights the diagnostic challenges of recognizing ABPA in children with difficult-to-treat asthma.

## Case report

An 11-year-old girl presented in July 2024 with persistent cough for two months. She had a 5-year history of asthma, definitively diagnosed at a tertiary teaching hospital based on typical variable respiratory symptoms and positive bronchial dilation test. She was prescribed combination therapy with inhaled corticosteroids (ICS) and long-acting beta_2_-agonists (LABA). Despite good adherence and proper inhaler technique verified by the attending physician, her asthma remained poorly controlled. Genetic testing was performed to rule out primary immunodeficiency, cystic fibrosis, and other related disorders. She had comorbid allergic rhinitis but no family history of asthma or other atopic diseases. The patient resided in Taizhou, Zhejiang Province, China, a coastal region with abundant rainfall and high humidity, which suggested potential mold exposure. During the preceding 12 months, she had been diagnosed with pneumonia on three separate occasions, each presenting with cough and wheezing, and had received multiple courses of antibiotics and short-term corticosteroids. Although these interventions produced transient symptomatic amelioration, respiratory symptoms persisted.

**Figure 1:**
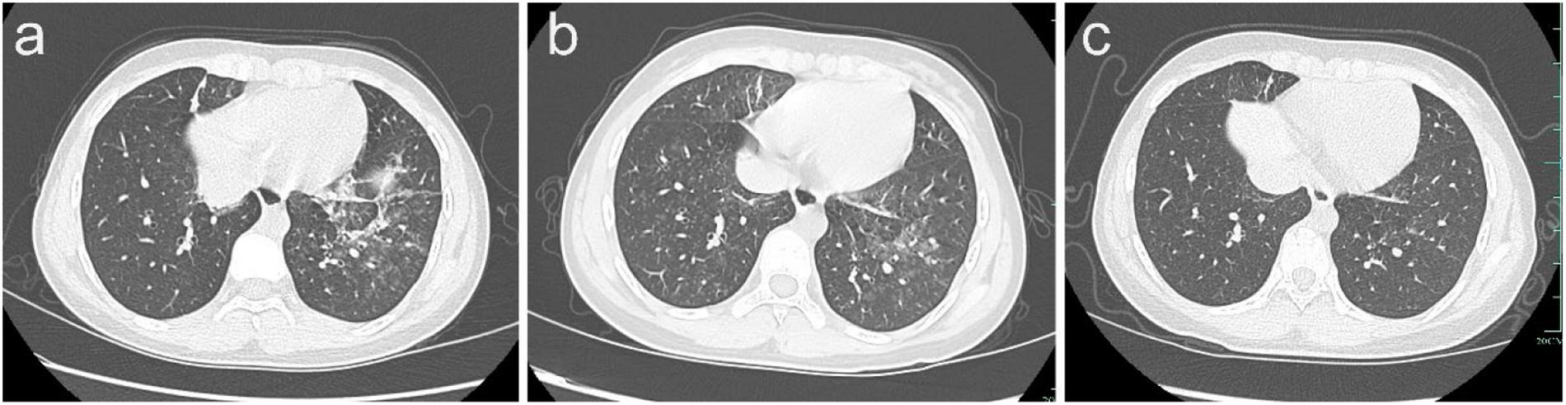
Chest high-resolution computed tomography: **(a)** May, 2024 (pre-treatment): bilateral pulmonary infiltrates. **(b)** July, 2024 (at admission): bilateral infiltrates with scattered micronodules. **(c)** December, 2024 (following corticosteroid and itraconazole therapy): marked resolution of pulmonary infiltrates.

Two months prior to admission, outpatient chest computed tomography demonstrated pulmonary infiltrates (Figure 1a), and she was again diagnosed with pneumonia. She was treated sequentially with intravenous amoxicillin-clavulanate for three days, ceftriaxone plus methylprednisolone for five days, and azithromycin for five days. Despite modest transient improvement, intermittent cough persisted. One day before admission, she presented with escalating cough and wheeze, prompting hospitalization and comprehensive diagnostic evaluation. On admission, vital signs were stable. Physical examination revealed scattered wheezes in both lungs, with no rales, no cyanosis of the lips or nails, no lymphadenopathy, regular heart rhythm without murmurs, and warm extremities without edema.

**Figure 2:**
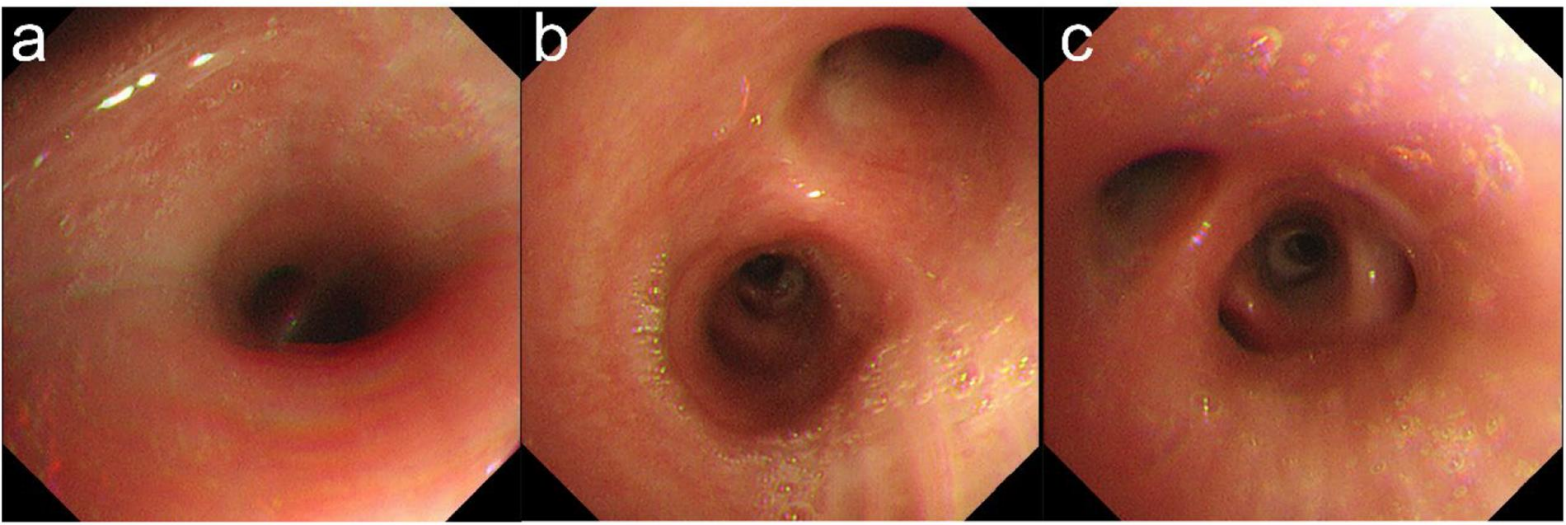
Bronchoscopic findings. **(a)** Main trachea: hyperemic and edematous mucosa with copious yellowish-white secretions. **(b,c)** Left and right bronchi, respectively: hyperemic and edematous mucosa with copious yellowish-white secretions.

Laboratory investigations revealed elevated total serum immunoglobulin E (IgE) at 678 IU/mL and *Aspergillus fumigatus*-specific IgE of 5.81 kUA/L (positive threshold > 0.35 kUA/L). Peripheral eosinophil count was 0.23 × 10⁹/L (within normal limits) on current admission; however, historical records documented eosinophilia > 0.5 × 10⁹/L on multiple occasions within the preceding year. High-resolution computed tomography (HRCT) demonstrated bilateral pulmonary infiltrates with scattered micronodules (Figure 1b). Bronchoscopy revealed copious purulent secretions coating the mucosa of the main trachea and bilateral bronchi (Figures 2a–c). Bronchoalveolar lavage fluid (BALF) cultures yielded *Aspergillus* species, and targeted next-generation sequencing (tNGS) of BALF detected *Aspergillus fumigatus*.

The patient, with a documented history of asthma as the underlying predisposing condition, fulfilled two mandatory diagnostic criteria: *Aspergillus fumigatus*-specific IgE ≥ 0.35 kUA/L and serum total IgE ≥ 500 IU/mL. Additionally, two of the three ancillary criteria were satisfied: (i) peripheral blood eosinophil count ≥ 500 cells/µL (could be historical), and (ii) pulmonary infiltrates on chest imaging—specifically, fleeting opacities documented across three sequential chest radiographs performed during pneumonia episodes over the preceding year—despite the absence of bronchiectasis, mucus plugging, or high-attenuation mucus on thoracic computed tomography. A diagnosis of allergic bronchopulmonary aspergillosis (ABPA) was therefore established (Table 1). Differential diagnoses are presented in Table 2. The patient was initiated on combination therapy: itraconazole 200 mg administered twice daily for four months, and oral prednisone 0.5 mg/kg/day for two weeks, followed by alternate-day dosing at equivalent intensity for eight weeks, with subsequent tapering by 2.5–5.0 mg increments every two weeks. Total corticosteroid duration was four months. Itraconazole is a broad-spectrum azole antifungal agent with good activity against *Aspergillus fumigatus*. It is widely used in pediatric ABPA treatment with established safety and efficacy data, readily available in our center, and cost-effective, making it suitable for long-term treatment (4 months) in this patient. Therapeutic drug monitoring of itraconazole serum levels was not available at our center; however, liver function was monitored monthly and remained within normal ranges throughout treatment. Itraconazole may potentiate the effects of corticosteroids when used concomitantly; however, no additional dosage adjustments of corticosteroids were required, and ICS-LABA therapy was continued for asthma control. No adverse events related to itraconazole or corticosteroids were observed during the entire treatment course, and the patient tolerated the therapeutic regimen well.

**Table 1:** Structured diagnostic assessment based on modified ISHAM criteria (2024) for the patient.

Diagnostic Domain	Specific Criterion (Modified ISHAM Criteria, 2024)	Patient Status	Category
Basic diseases	In patients with predisposing conditions (asthma, cystic fibrosis, chronic obstructive lung disease, bronchiectasis) or a compatible clinico-radiological presentation	5-year history of asthma	Met
Essential components	*A. fumigatus*-specific IgE ≥ 0.35 kUA/L	5.81 kUA/L	Met
	Serum total IgE ≥ 500 IU/mL	678 IU/mL	Met
Other components (any two)	Positive IgG against *A. fumigatus*	Not assessed	Not assessed
	Peripheral blood eosinophil count ≥ 500 cells/µL (could be historical)	Historical: > 0.5 × 10⁹/L; Current: 0.23 × 10⁹/L	Met
	Thin-section chest computed tomography consistent with ABPA (bronchiectasis, mucus plugging, and high-attenuation mucus) or fleeting opacities on chest radiograph consistent with ABPA	Fleeting opacities were observed on chest radiograph consistent with ABPA, whereas chest CT showed no bronchiectasis, mucus plugging, or high-attenuation mucus.	Met
Supportive (Non-Standard)	Bronchoscopy is recommended to obtain respiratory samples for fungal culture in patients with suspected allergic bronchopulmonary mycosis (ABPM) who have diagnostic uncertainty or uninformative or unobtainable sputum cultures.	The BALF culture was positive for *Aspergillus* species, and tNGS detected *A. fumigatus*	Supportive

**Table 2:** Differential diagnosis and key exclusion features.

Diagnosis	Key Distinguishing Features	Exclusion Basis in This Patient
Recurrent bacterial pneumonia	Fixed lobar consolidation; positive cultures; leukocytosis	Negative cultures; fleeting opacities consistent with ABPA; no leukocytosis
*Aspergillus* colonization	Asymptomatic; absent specific IgE; normal imaging	Symptomatic disease; specific IgE 5.81 kUA/L; bilateral infiltrates
Eosinophilic lung disease	Peripheral infiltrates; sustained eosinophilia > 1.5 × 10⁹/L	Normalized eosinophils; *Aspergillus* sensitization
Pulmonary tuberculosis	Cavitation; tree-in-bud; T-SPOT/culture positive	Negative T-SPOT; no cavitation; no lymphadenopathy
Atypical mycobacterial infection	Bronchiectasis with nodules; positive AFB culture	Negative BALF tNGS; absent bronchiectasis; normal immunoglobulins
Cystic fibrosis	Sweat chloride ≥ 60 mmol/L; CFTR mutations	CFTR negative

The girl responded well to the therapy. Cough and wheezing resolved completely within three weeks. At one-month follow-up, total IgE had decreased to 479 IU/mL, representing a 29% reduction from baseline. Follow-up HRCT at four months showed substantial resolution of pulmonary infiltrates (Figure 1c). No recurrence of cough or wheezing was observed during treatment or after discontinuation. Following completion of the 4-month ABPA treatment, the patient did not return for scheduled clinic visits; repeated pulmonary function testing and serial IgE measurements were therefore not performed. Telephone follow-up at approximately one year confirmed the patient remained asymptomatic without cough or wheezing. The clinical timeline of the patient is shown in Table 3.

**Table 3:** Clinical timeline of the patient.

Timepoint	Clinical Event	Key investigations	Treatment
8 months before admission (December 2023)	First episode of pneumonia, presenting with fever, cough, and wheezing	Chest x-ray showed fleeting opacities	Antibiotics, ICS + LABA
4 months before admission (March 2024)	Second episode of pneumonia, presenting with cough and wheezing	Chest x-ray showed fleeting opacities	Antibiotics, ICS + LABA
2 months before admission (May 2024)	Third episode of pneumonia, presenting with fever, cough, and wheezing	Chest x-ray showed fleeting opacities	Antibiotics, short-term corticosteroids, ICS + LABA
Admission (July 2024)	Persistent cough for 2 months	1.Total IgE: 678 IU/mL; *A. fumigatus*-specific IgE: 5.81 kUA/L; 2. HRCT: Bilateral infiltrates + micronodules; 3. BALF culture and tNGS: Positive for *A. fumigatus*	Itraconazole + Prednisone, ICS + LABA
1 month after treatment initiation (August 2024)	No cough or wheezing	1. Total IgE: 479 IU/mL (29% decrease from baseline); 2. Liver function: Within normal ranges	Itraconazole + Prednisone (tapering), ICS + LABA
4 months after treatment initiation (end of treatment)	No cough or wheezing	1. HRCT: marked resolution of pulmonary infiltrates.2. Liver function: Within normal ranges	ICS + LABA

The patient and her guardian stated that they had suffered significant distress and anxiety prior to the definitive diagnosis, owing to recurrent pneumonia episodes and multiple courses of antibiotic therapy. They noted that the correct diagnosis and targeted treatment brought considerable relief, and the patient was able to return to normal school activities within one month of treatment initiation. They were highly satisfied with the clinical outcome.

The patient provided written informed consent for both the medical treatment and the publication of this case report. The study was approved by the Ethics Committee of Taizhou Municipal Hospital (Taizhou University Affiliated Municipal Hospital), School of Medicine (Approval No. 202600331).

## Discussion

Allergic bronchopulmonary aspergillosis (ABPA), first described in 1952 by Hinson et al. (5) and first reported in a pediatric patient in 1959 (6), represents a severe hypersensitivity pulmonary disease triggered by *Aspergillus fumigatus* colonization. While classically complicating asthma and cystic fibrosis (CF), ABPA increasingly emerges in the context of bronchiectasis secondary to tuberculosis, primary ciliary dyskinesia, and immunodeficiencies such as chronic granulomatous disease and hyper-IgE syndrome (7, 8). Disease progression may lead to recurrent exacerbations, bronchiectasis, massive hemoptysis, pulmonary fibrosis, chronic type II respiratory failure, and cor pulmonale. Notably, 4%–10% of ABPA patients ultimately develop chronic pulmonary aspergillosis (CPA) (4), indicating that early recognition and intervention are important to reduce the risk of irreversible lung damage. *Aspergillus* sensitization (AS) is defined as immediate cutaneous hypersensitivity to *Aspergillus fumigatus* antigens or elevated levels of *Aspergillus fumigatus*-specific IgE. Allergic bronchopulmonary aspergillosis (ABPA) represents an advanced phenotype of *Aspergillus* sensitization, and AS constitutes the initial pathogenic step in the development of ABPA. A recent meta-analysis reported a pooled prevalence of *Aspergillus fumigatus* sensitization of 25% among adult patients with asthma in tertiary care centers. Approximately 37% of individuals with *Aspergillus* sensitization may progress to ABPA. Prior literature has therefore suggested routine assessment for *Aspergillus fumigatus* among adults with asthma evaluated at tertiary medical institutions. The 2013 diagnostic criteria proposed by the ISHAM ABPA Working Group (AWG) have long served as the clinical benchmark; however, evolving evidence regarding diagnostic test performance has necessitated revision. The 2024 ISHAM clinical practice guidelines now recommend screening for *Aspergillus*-specific IgE exclusively in children with difficult-to-treat asthma rather than all asthmatic patients (9, 10). Yet, despite this refinement, pediatric ABPA lacks standardized, unified diagnostic pathways, which may contribute to missed or delayed diagnosis.

Our case illustrates these challenges. The patient's three pneumonia misdiagnoses within a single year precisely mirror published data indicating that 30% of pediatric ABPA cases are initially misdiagnosed as bacterial pneumonia or tuberculosis (1), exposing a critical deficit in frontline clinical recognition. This diagnostic delay may relate to three interlocking challenges. First, clinical manifestations lack specificity. The absence of expectorated brownish, gelatinous sputum—a symptom significantly less frequent in children due to impaired airway clearance (11)—combined with the lack of characteristic radiographic features such as bronchiectasis or high-attenuation mucus (HAM) plugs, may create a false reassurance. Although a 2020 review acknowledged that ABPA may present without radiographic abnormalities and emphasized that the diagnosis is primarily immunological (1), clinical practice may still over-rely on imaging while undervaluing immunological clues. Second, corticosteroid therapy may mask key laboratory parameters. The patient's admission eosinophil count was normal, but historical data documented prior values exceeding 0.5 × 10⁹/L. The 2024 ISHAM guidelines explicitly endorse using historical eosinophilia (9), which was relevant in this case because corticosteroid exposure may suppress eosinophil counts and produce a misleading normalization (1). Third, diagnostic thresholds remain outdated at the bedside. Our patient's total IgE of 678 IU/mL, although below the conventional 1,000 IU/mL cutoff, satisfied the revised 2024 ISHAM threshold of ≥ 500 IU/mL—a modification that increased sensitivity from 91% to 98% without compromising specificity (9). This case is therefore consistent with the potential value of updated diagnostic thresholds in reducing underdiagnosis.

Although the patient met core criteria (asthma + required markers: total IgE ≥ 500 IU/mL, positive *Aspergillus*-specific IgE), she lacked other supportive features (radiographic hallmarks, IgG not tested), rendering her an atypical case. However, *Aspergillus* species were cultured from bronchoalveolar lavage fluid, and tNGS detected *Aspergillus fumigatus* sequences, providing supportive microbiological evidence. While current guidelines have yet to formally integrate BALF culture or tNGS into ABPA diagnostic algorithms, the 2024 ISHAM standards already acknowledge a single positive fungal culture from BALF as contributory evidence in allergic bronchopulmonary mycosis (ABPM) diagnosis (9). Moreover, a Chinese retrospective study employing metagenomic next-generation sequencing demonstrated that ABPA patients with *Aspergillus fumigatus* airway colonization exhibited more severe disease and higher exacerbation risk (12). With the widespread application of gene sequencing technologies, the value of tNGS in the diagnosis of ABPA warrants further in-depth investigation and validation.

The favorable clinical and biomarker response was consistent with the diagnosis of ABPA. Following itraconazole plus corticosteroid initiation, symptom resolution, marked radiographic clearance, and a 29% total IgE reduction (to 479 IU/mL) after one month exceeded the > 20% decline threshold defining treatment response in the 2024 ISHAM guidelines (9). This dynamic biomarker shift, viewed through a therapeutic lens, supports the clinical impression of ABPA in this patient. Notably, treatment success also hinged on timely antifungal initiation; itraconazole (200 mg twice daily) was selected based on established pediatric experience, availability, and cost-effectiveness, achieving excellent outcomes when combined with corticosteroids for four months.

Over the past two decades, accumulating evidence has supported the use of monoclonal antibodies for the treatment of ABPA. Agents including omalizumab, mepolizumab, benralizumab, and dupilumab have been applied in patients with ABPA. Among biological agents, the majority of clinical experience in ABPA is with omalizumab. Use of omalizumab in ABPA has been associated with improved clinical symptoms, reduced acute exacerbations and asthma-related hospitalizations, enhanced pulmonary function, and decreased oral corticosteroid dosage (9). However, current studies are limited by the lack of placebo-controlled designs, and further prospective clinical trials are warranted to evaluate the long-term efficacy of omalizumab or other anti-IgE monoclonal antibodies in ABPA.

This case also highlights unresolved issues in pediatric ABPA management, particularly the continued reliance on adult-derived criteria in children. However, as a single case without complete long-term objective follow-up, our report should be interpreted cautiously. Future multicenter prospective studies are needed to refine pediatric diagnostic thresholds—particularly for total IgE and eosinophil counts—and to further evaluate the diagnostic contribution of emerging technologies such as tNGS. In clinical practice, *Aspergillus*-specific IgE screening may be considered in children with difficult-to-treat asthma, particularly when recurrent pulmonary infiltrates or recurrent pneumonia are present. Historical laboratory trends, microbiological evidence, and treatment response may also assist diagnostic assessment.

## Conclusion

This case highlights potential diagnostic challenges in atypical pediatric ABPA, suggesting the importance of heightened clinical suspicion for ABPA in children with difficult-to-treat asthma and recurrent pneumonia. At its core, widespread underdiagnosis may relate to the absence of pediatric-specific criteria and passive, rather than proactive, screening protocols. We therefore suggest screening for *Aspergillus*-specific IgE in children with difficult-to-treat asthma, aligning with the 2024 ISHAM guidelines that endorse simplified diagnostic workflows, reduced total IgE thresholds, and amplified recognition of asthma-ABPA comorbidity. Future multicenter prospective cohorts are needed to establish pediatric-specific diagnostic cutoffs—particularly for total IgE and eosinophil counts—to rectify this evidence gap. Ultimately, early diagnosis and swift intervention may help reduce the risk of irreversible pulmonary damage.

## Data Availability

The raw data supporting the conclusions of this article will be made available by the authors, without undue reservation.
